# The relevance of stretch intensity and position—a systematic review

**DOI:** 10.3389/fpsyg.2015.01128

**Published:** 2015-08-18

**Authors:** Nikos Apostolopoulos, George S. Metsios, Andreas D. Flouris, Yiannis Koutedakis, Matthew A. Wyon

**Affiliations:** ^1^Research Centre for Sport, Exercise and Performance, Institute of Sport, University of WolverhamptonWalsall, UK; ^2^Department of Exercise Sciences, University of ThessalyTrikala, Greece; ^3^National Institute of Dance Medicine and ScienceLondon, UK

**Keywords:** stretching, intensity, inflammation, performance, injury, rehabilitation

## Abstract

Stretching exercises to increase the range of motion (ROM) of joints have been used by sports coaches and medical professionals for improving performance and rehabilitation. The ability of connective and muscular tissues to change their architecture in response to stretching is important for their proper function, repair, and performance. Given the dearth of relevant data in the literature, this review examined two key elements of stretching: stretch intensity and stretch position; and their significance to ROM, delayed onset muscle soreness (DOMS), and inflammation in different populations. A search of three databases, Pub-Med, Google Scholar, and Cochrane Reviews, identified 152 articles, which were subsequently categorized into four groups: athletes (24), clinical (29), elderly (12), and general population (87). The use of different populations facilitated a wider examination of the stretching components and their effects. All 152 articles incorporated information regarding duration, frequency and stretch position, whereas only 79 referred to the intensity of stretching and 22 of these 79 studies were deemed high quality. It appears that the intensity of stretching is relatively under-researched, and the importance of body position and its influence on stretch intensity, is largely unknown. In conclusion, this review has highlighted areas for future research, including stretch intensity and position and their effect on musculo-tendinous tissue, in relation to the sensation of pain, delayed onset muscle soreness, inflammation, as well as muscle health and performance.

## Introduction

Stretching refers to a movement applied by an external and/or internal force in order to increase one's joint range of motion i.e., flexibility (Light et al., 1984; Weerapong et al., 2004). Forms of stretching include active, passive, dynamic, static, ballistic, and proprioceptive neuromuscular facilitation (PNF) (Sady et al., 1982; Shellock and Prentice, 1985; Alter, 2004; Bonnar et al., 2004; Shrier, 2004). Traditionally, stretching exercises have been advocated by sports coaches and medical professionals as a means for performance enhancement and injury prevention by regaining joint range of motion (ROM) i.e., increasing flexibility (Hortobágyi et al., 1985; Taylor et al., 1990; Wilson et al., 1991).

Stretching depends on the active and passive tension of the muscle, the musculo-tendinous unit (MTU), as well as the proprioceptors of the musculoskeletal system, the muscle spindles, and the Golgi tendon organs (Nikolaou et al., 1987; Guissard and Duchateau, 2006; Knudson, 2006; Abdel-aziem et al., 2013). The tension created by muscle can be classified as either active or passive, with active referring to the interaction of the actin and myosin filaments of muscle, and passive to the elongation of the connective tissue beyond their resting length (Knudson, 2006). Both active and passive define the length-dependent properties of muscle which is strongly related to stretching, for the interaction of each implies that exercise interventions, like stretching, may have a complex effect on skeletal muscle, dependent on the interaction of the tissues and the nature of the training stimulus (Knudson, 2006). In other words, when muscle is stretched using stretching techniques [i.e., static, active, dynamic, or PNF] these may account for changes in the active and passive tension of muscle improving the ROM about a joint (Knudson, 1999).

The MTU features prominently in stretching, with Kubo et al. (2001) suggesting the potential mechanism for reduced risk of injury with increased flexibility is the change in its viscoelastic properties. During stretching, with the MTU being held at a constant length, the passive force at that length gradually declines, resulting in a stress relaxation (Magnusson et al., 1995). *In-vivo* (Magnusson et al., 1995) and *in-vitro* (Taylor et al., 1990) studies have observed that repeated stretching of the MTU to a constant length reduces peak passive tension, suggesting that this reduction in the viscosity and/or stiffness of the MTU during stretching is responsible for the increase in the joint ROM (Kubo et al., 2001).

Within the muscle fibers and tendons are located the proprioceptors, sensors providing information about joint angle, muscle length, and muscle tension. Two proprioceptors related to stretching are the muscle spindles (respond to changes in length) and the Golgi tendon organs (respond to changes in tension) (Guissard and Duchateau, 2006; Abdel-aziem et al., 2013), relaying information about muscular tension to the central nervous system (Abdel-aziem et al., 2013). Therefore, the interplay of muscle tension (active and passive), the MTU and viscoelasticity and the proprioceptive tissue (muscle spindles and Golgi tendon organ), are important when considering how stretching may influence the increase or decrease of flexibility and the ROM about a joint.

In the literature, four stretch parameters have been identified as being important for potentially influencing the increase or decrease of flexibility of a joint: intensity, duration, frequency (Marschall, 1999), and stretch position (Wyon et al., 2009). The focus of this review was on the intensity of the stretch and stretch position. Intensity is important for the magnitude of force generated during stretching may influence the response of the tissue. For instance, too little force may result in an elastic response with little or no gain in ROM (Jacobs and Sciascia, 2011), while the application of too much force may injure the tissue, leading to an inflammatory response (Brand, 1984; McClure et al., 1994). The rationale for including stretch position is that this may directly or indirectly influence the intensity of the stretch, for muscle and tendon tissue and their components (i.e., collagen) are known to respond to altered levels of activity (Kjaer, 2004). The position assumed during stretching may influence the magnitude of the force generated prior to and during the stretch potentially altering the response of the muscle and tendon tissue. In a study by Abdel-aziem et al. (2013), comparing a standing hamstring stretch to a supine lying stretch, the supine lying stretch isolated the hamstring muscle better, was more comfortable, but more importantly facilitated a better relaxation response during the stretch. In this review, four positions were identified: loaded, supported, therapist, and machine, with each being defined in the Materials and Methods Section (*stretching intensity and position*). Though joint angle, force direction, magnitude, and duration of stretch may remain identical, this does not preclude the notion that a force might be generated in relation to the stretch position heightening the stress or strain on the muscle, tendon, and the MTU. Even though the force generated during the stretch on the muscle and tendon tissue is not known with regard to different stretch positions, suggesting further research, what is known and has been observed is that the load imparted by force affects the structural and functional adaptation of the tissue (Kjaer, 2004).

This adaptation of muscle to force refers to muscle plasticity, a mechanical property suggesting the ability of muscle cells to alter their structure and function in response to various stimuli (Martins et al., 2013). It has been observed that the stretch of muscle cells interact closely with skeletal muscle tissue suggesting an adaptive process when subjected to a mechanical load (Kjaer, 2004). Load has been defined as a either a cyclic or static stretch, strain or shear stress, with a combination of these loads being responsible for altering the shape of a body resulting in an adaptation (Salameh and Dhein, 2013). These forces can deform the extracellular matrix (ECM), which links tissues of the body together playing an important role in the tissue structure maintenance of tendons, ligaments and muscle (Kjaer, 2004). Studies on stretching have indicated that stretching can promote sarcomeregenesis, a synthesis of contractile protein produced by specific muscle, by machanotransduction (Martins et al., 2013). During stretching, this mechanical stimulus affects the ECM, with the integrins, the transmembrane receptors bridging cell-ECM interactions, detecting and transmitting this stimulus into the cell interior (De Deyne, 2001). This stimulus activates a series of nuclear proteins modifying gene transcription regulating sarcomeregenesis (De Deyne, 2001). With intensity defined as the magnitude of force or torque being applied to the joint during a stretching exercise (Jacobs and Sciascia, 2011), and stress relaxation refers to a decrease in the force necessary to hold a tissue at a particular length over time, the combination of intensity and stretch position may play a significant role in increasing the ROM about a joint, possibly through the process of sarcomeregenesis. Subsequently, the combination of stretch intensity and position with duration and frequency may play a significant role in increasing ROM (Wyon et al., 2009, 2013), possibly influencing the body's response with regard to delayed onset muscle soreness (DOMS) or inflammation (Smith et al., 1993).

DOMS, is a sensation of dull, aching pain, combined with tenderness and stiffness occurring 24 h post unaccustomed exercise, peaking 1–3 days, disappearing by 7–10 days (MacIntyre et al., 1995). It is generally accepted that DOMS is associated with muscle and/or connective tissue damage, and/or subsequent inflammatory responses induced by eccentric exercise (Nosaka et al., 2002). According to Smith (1991) the observed events associated with acute inflammation are also seen with DOMS: swelling, loss of function, and pain. The symptoms and signs arising from normal tissue exposed to high intensity stimuli generally reflect the intensity, localization, and timing of the initiating stimuli (Kidd and Urban, 2001). Stretch intensity has been inherently mediated by pain, with stretching beyond the pain threshold for prolonged periods associated with an inflammatory response (Jacobs and Sciascia, 2011). Given the relationship of pain to tissue damage, and its relationship to inflammation (Merskey and Bogduk, 1994), it is very interesting that, although duration and frequency have attracted scientific attention, the magnitude of the stretch intensity and the body's position during stretching have not attracted as much.

It has been observed in the literature that the independent variables of duration and frequency, being “quantitative” in nature, are used extensively (Tables 1–**4**). They are probably easier to manipulate with participants instructed to hold a particular stretch for a certain length of time (duration) repeated for several sets (frequency). However, the independent variables of stretch intensity and position are more difficult to manipulate. They are “ordinal” in nature referring to a feeling, a perception unique to each participant. Experimenters often resort to descriptive terms to convey the sensation, what the intensity should feel like during the stretching exercise (i.e., discomfort, pain etc.), and the position adopted during the exercise (i.e., standing vs. a supine position). Therefore, given the difficulty manipulating stretching intensity and position, most articles mention them in conjunction with duration and frequency relative to applications both clinically and athletically. To date there are no systematic reviews focused on stretch intensity and body position and how this may affect the soft and connective tissue. This is interesting since stretch intensity and body position have been included in the design of stretching experiments presuming their relevance, however they have not been fully investigated. In fact, most systematic reviews refer to stretching in response to muscle performance (Weerapong et al., 2004; Rubini et al., 2007; Kay and Blazevich, 2012), muscle soreness and injury risk (Herbert and Gabriel, 2002; Connolly et al., 2003; Thacker et al., 2004), and increases in ROM (Decoster et al., 2005; Harvey et al., 2006).

**Table 1:** **Athlete population**.

**Article**	***n***	**Methodology**				**Main outcomes**	**Q**
		**MeSH term**	**Type of stretch**	**Elements of stretching**	**Stretch position**		
Hayes and Walker, 2007^‡^	7	ROM	Static dynamic	Intensity D, duration, frequency	Loaded	Pre-exercise stretching no impact on running economy or sub-max exercise oxygen cost.	3
Silveira et al., 2011^‡^	12	ROM	Static dynamic	Intensity D duration frequency	Loaded	Static stretching did not improve dynamic hamstring flexibility; however, dynamic stretching improved both dynamic and static flexibility.	3
Wyon et al., 2013^‡^	39	ROM	Static	Intensity G, Dduration, frequency	Supported	Low intensity stretching beneficial in development of active and passive ROM.	3
Allison et al., 2008^†^	10	ROM	Static	Intensity D, duration, frequency	Loaded	Prolonged static stretch no influence on running economy despite changes in neuromuscular function.	2
Ayala et al., 2010	18	ROM	Active	Duration, frequency	Loaded	Effect of acute stretching prior to intensive activity needs to be considered before pre-exercise warm up routine.	1
Bazett-Jones et al., 2008^†^	21	ROM	Static	Intensity D, duration, frequency	Loaded	After 6 weeks static stretch hamstring no improvement in knee ROM, sprint or vertical jump.	1
Bazett-Jones et al., 2005	10	ROM	Static	Duration, frequency	Therapist	Potentiating effect ↑ ROM but also fatigue.	1
Bello et al., 2011	14	ROM	Passive	Duration, frequency	Therapist	Athletes Rhythmic stabilization >Passive Stretch fewer injuries.	1
Caplan et al., 2009^†^	18	ROM	Static PNF	Intensity D, duration, frequency	Loaded	Both static stretch and PNF ↑ Hip Flexor ROM, running mechanics during high velocity running.	1
Decicco and Fisher, 2005	30	ROM	PNF	Duration, frequency	Therapist	CRC, HRC PNF ↑ external shoulder ROM: 2×/week for 6 weeks.	1
Favero et al., 2009^†^	10	ROM	Static	Intensity P, duration, frequency	Loaded	Acute stretching no conclusive evidence on sprint performance in the context of the athletes existing ROM.	1
Halbertsma et al., 1996	16	ROM	Static	Duration, frequency	Loaded	1–10 min stretch ↑ passive muscle moment ROM elongation of hamstring.	1
Herman and Smith, 2008	24	ROM	Static dynamic	Duration, frequency	Loaded	4 weeks of dynamic warm up intervention daily preseason ↑ sustained power, strength, muscle endurance anaerobic, and agility.	2
Larsen et al., 2005	20	ROM	Static	Duration, frequency	Loaded	Static stretch no effect on knee Joint Position Sensation.	2
Maenhout et al., 2012^†^	62	ROM	Static	Intensity G, duration, frequency	Loaded	Acromiohumeral distance smaller on dominant side in athletes with glenohumeral internal rotation deficit. This 2-D measure of subacromial space was found to ↑ after 6 week sleeper stretch.	2
Magnusson et al., 1998^†^	12	ROM	Static	Intensity P, duration, frequency	Machine	Static stretch and cycling stretch ↑ joint ROM by ↑ stretch tolerance.	1
McBride et al., 2007	8	ROM	Static	Duration, frequency	Therapist	Stretching ↓ muscle force output in single joint isometric contraction and rate of force development in multiple joint isometric contraction.	1
Mendez-Sanchez et al., 2009	8	ROM	Static active	Duration, frequency	Therapist	Adding sciatic nerve slider to sustained hamstring stretching ↑ in both lumbar and lower quadrant flexibility.	2
Moller et al., 1985	48	ROM	PNF	Duration, frequency	Loaded	ROM training ↓ post 24 h, stretching prior to activity, ↑ knee flexion, post training greatest ↑ hip extension flexion and knee flexion.	1
Morrin and Redding, 2013^†^	10	ROM	Staticballistic	Intensity MSNP, duration, frequency	Loaded	It has been suggested that a combined warm-up protocol consisting of SS and DS should be promoted as an effective warm-up for dancers.	1
Roberts and Wilson, 1999^†^	12	ROM	Static	Intensity D, duration, frequency	Loaded	15 s stretch > 5 s ↑ improvements in active ROM.	1
Torres et al., 2008	11	ROM	Static dynamic	Duration, frequency	Loaded	No short-term effect of stretching on upper-body muscular performance regardless of stretch mode.	2
Tsolakis et al., 2010^†^	20	ROM	Staticdynamic	Intensity G, duration, frequency	Loaded	Static or ballistic stretching in the later stages of a general warm-up normally used before training or competition does not hinder specific performance in fencing.	1
Zourdos et al., 2012	14		Dynamic	Duration frequency	Loaded	Dynamic stretching does not affect running endurance performance in trained male runners.	1

MeSH, medical search headings; ROM, range of motion; PNF, proprioceptive neuromuscular facilitation; D, discomfort; P, pain; G, gentle; ↑, increased; ↓, decreased; Q, quality of study;

† Indicates study mentioning intensity;

‡ *Indicates high quality study mentioning intensity*.

With this literature review four populations were chosen (athlete, clinical, elderly, and general) each highlighting a variance with the use of and importance of stretching. It should be noted that the general population referenced studies that do not specifically refer to athletes, clinical, and the elderly. Within the athletic group, athletes, coaches, and trainers recommend stretching in an effort to both prevent injury and enhance performance (Thacker et al., 2004). In the clinical population, stretching is used to deal with numerous pathophysiological conditions such as; stroke, contractures, and various musculoskeletal disorders in order to provide relief from pain. With the elderly population, the greatest concern with stretching is increasing the movement of the lower limb in order to improve gait and mobility (Christiansen, 2008; Cristopoliski et al., 2009). However, though these variances amongst these populations are important, the emphasis of this review is to investigate how stretch intensity and body position may impact and influence the soft and connective tissue of these populations, given the dearth of studies with regard to these parameters of stretching.

## Materials and methods

**Figure 1:**
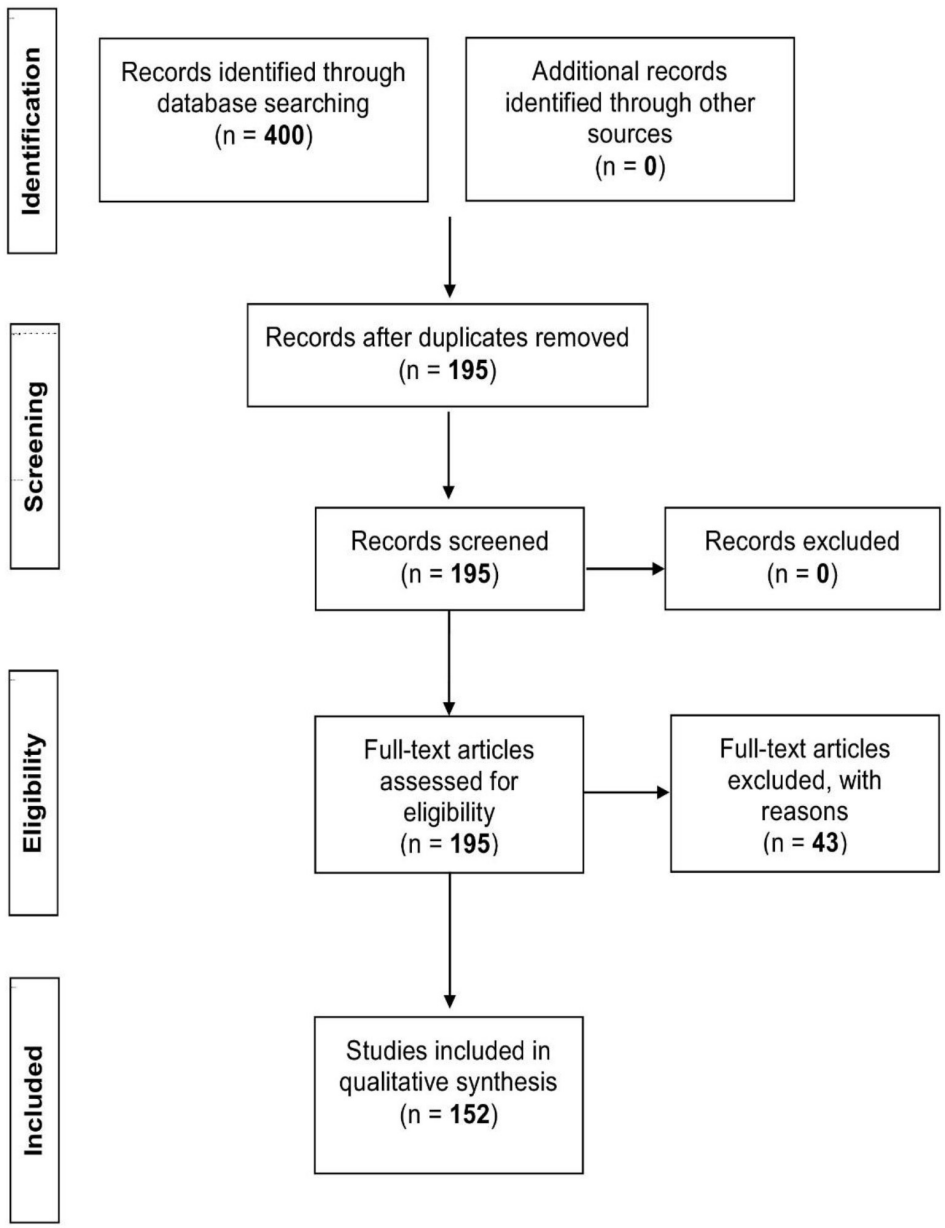
**PRISMA flow diagram**.

Using the following limits: humans (adults), English language, clinical trials, randomized controlled trials, and reviews, three databases were consulted (PubMed/Medline, Google Scholar, and Cochrane reviews), in order to determine stretch intensity and its association to stretching. The Medical Subject Heading (MeSH) terms, “stretch” and “stretching exercises,” were utilized in combination with “ROM,” “DOMS,” and “inflammation” in four different populations: athletes, clinical, elderly, and general population. The athlete group consisted of all studies mentioning an athletic activity which included only athletes, the clinical group concerned patients with any disease/injury, and the elderly group included individuals of 65+ years of age. The general group comprised all remaining studies not adhering to the criteria set for the three aforementioned groups. In addition, a “*quality of study*” assessment as set out by Jadad et al. (1996) was employed to evaluate the quality of the selected studies. Eligibility for each study was based on the following criteria. If studies were described as randomized they were given a point, with a further point awarded if the method of randomization was described. Randomization was deemed appropriate if it allowed each participant to have the same access of receiving the intervention, if this criteria was not met a point was deducted. Studies were also given a point if they were described as double blind, with another point assigned if both the person administering and the participant receiving the intervention could not identify it. If this criteria was not met a point was deducted. In turn, a point was given if the study described the number and the reasons for participant withdrawal. The maximum score for each study was five with the minimum for an included report being one. Scores of three or greater were deemed of high quality. Studies that did not meet any of the criteria were excluded from the literature review. The initial search produced 400 relevant articles, of which 205 overlapped between databases, leaving 195 studies. Of these studies, 43 were excluded for not satisfying any of the criteria of the “quality of study” assessment as described above. This resulted in the inclusion of 152 articles (Athlete *n* = 24; Clinical *n* = 29; Elderly *n* = 12; General *n* = 87) for this review (Figure 1, PRISMA flow diagram).

### Stretching intensity and position

For the purpose of this literature review, the stretch intensity for each article was assessed if it referenced to a subjective sensation of the stretch: discomfort, gentle (feeling of gentle pull on the muscle), maximum stretch no pain (MSNP), and pain, or mentioned the use of a scale (i.e., numerical, visual, and soreness rating scale). In turn, position was assessed by assessing the execution of the stretching exercise as either being: supported, loaded, therapist, and machine assisted. Supported referred to the placement of the body in a position where it is stable with a broad base of support (i.e., lying down vs. standing up). A loaded stretch, pertained to placing the body in a stretch position where the muscle that is being stretched is also called upon to help support the body during the stretch (i.e., a lying down hamstring stretch vs. a stand up hamstring stretch). The last two stretch positions referred to the use of a machine (e.g., isokinetic dynamometer) or a therapist as the means of applying the force on the muscle-tendon unit. The sensations and positions described above are conscious in nature (Proske and Gandevia, 2012), with the individual being aware of tension generated by the muscle during the stretching. This sensory input involved generates an awareness that may influence stretch intensity.

## Results

### Athlete population

Twenty-four studies were identified in which all mentioned duration and frequency, but only 12 referenced intensity (Magnusson et al., 1998; Roberts and Wilson, 1999; Hayes and Walker, 2007; Allison et al., 2008; Bazett-Jones et al., 2008; Caplan et al., 2009; Favero et al., 2009; Tsolakis et al., 2010; Silveira et al., 2011; Maenhout et al., 2012; Morrin and Redding, 2013; Wyon et al., 2013) (Table 1). Of these 12 studies, 11 had participants perform a loaded stretch of which six had them stretch to discomfort (Roberts and Wilson, 1999; Hayes and Walker, 2007; Allison et al., 2008; Bazett-Jones et al., 2008; Caplan et al., 2009; Silveira et al., 2011). It is interesting to note that two studies observed stretching had no impact on running economy (Hayes and Walker, 2007; Allison et al., 2008), one study noticed no improvement in knee ROM (Bazett-Jones et al., 2008), with another observing no improvement in dynamic hamstring flexibility (Silveira et al., 2011). However, this study did observe that dynamic stretching did improve both dynamic and static flexibility. Interestingly the two remaining studies indicated that stretching to discomfort resulted in a benefit with an increase in hip flexor ROM (Caplan et al., 2009) and active ROM (Roberts and Wilson, 1999). Such a contradiction suggests the need to perform more studies specifically looking at how stretching to discomfort during a loaded stretch may affect the musculoskeletal system. Two studies had participants stretch using a gentle intensity stretch (Tsolakis et al., 2010; Maenhout et al., 2012). Tsolakis et al. observed that gentle stretching used before training or competition did not hinder performance in fencing (Tsolakis et al., 2010), with Maenhout et al. noticing an increase in subacromial space after 6 weeks of stretching (Maenhout et al., 2012). Two studies which had participants stretch to pain used different stretch positions, loaded (Favero et al., 2009) and machine (Magnusson et al., 1998) with results indicating that acute stretching did not improve sprint performance (Favero et al., 2009) and an increase in ROM was due to stretch tolerance (Magnusson et al., 1998). This is in direct contrast to the studies which observed an improvement with the use of gentle intense stretching (Tsolakis et al., 2010; Maenhout et al., 2012). In turn, the study by Wyon et al. which referred to both a gentle and discomfort intense stretch with support, observed an increase in both active and passive ROM with use of a gentle stretch (Wyon et al., 2013).

It is worth highlighting, that within this population, only three studies were of high quality (Hayes and Walker, 2007; Silveira et al., 2011; Wyon et al., 2013) (Refer to Table 1), indicating the need to perform more studies in order to observe how intensity and body position may impact the athletic population.

### Clinical population

Twenty-nine studies have been conducted on clinical populations suffering from neck pain, cancer, continuous obstructive pulmonary disease, contractures, as well as joint and trigger point issues (Table 2). All 29 studies mentioned duration and frequency, with seven referencing stretch intensity(Light et al., 1984; Hanten et al., 2000; Horsley et al., 2007; Cunha et al., 2008; Maluf et al., 2010; Trampas et al., 2010; Renan-Ordine et al., 2011), of which only two were of high quality (Horsley et al., 2007; Maluf et al., 2010). These high quality studies had participants perform loaded stretches with Maluf et al. having patients stretch to discomfort, while Horsley et al. used a MSNP. Interestingly, the study by Horsley et al. observed no benefit with use of stretching with regard to wrist contracture of stroke patients, a central nervous system (CNS) issue, while Maluf et al. indicated a benefit of stretching for transmandibular pain, a peripheral nervous system (PNS) issue.

**Table 2:** **Clinical population**.

**Article**	***n***	**Methodology**				**Main outcomes**	**Q**
		**MeSH term**	**Type of stretch**	**Elements of stretching**	**Stretch position**		
Horsley et al., 2007^‡^	40	ROM	Static	Intensity MSNP, duration, frequency	Loaded	4 weeks of regular stretching little or no effect on wrist contracture after stroke, upper limb pain or improved activity.	3
Maluf et al., 2010^‡^	24	ROM	Static	Intensity D, duration, frequency	Loaded	Global postural re-education and static stretching both effective for Transmandibular ↓pain intensity, EMG activity ↑ pain thresholds.	3
Albayrak et al., 2014	36	ROM	Static	Duration, frequency	Therapist	Stretching exercise increased flexion mobility as a result both depression level and health status improved.	3
Gustafsson and McKenna, 2006	32	ROM	Static	Duration, frequency	Machine	Treatment group ↑ pain.	33
Häkkinen et al., 2007	125	ROM	Static	Duration, frequency	Therapist	Manual therapy and stretching effective short-term ↓ spontaneous strain pain in patients with chronic neck pain.	3
Harvey et al., 2000	14	ROM	Static	Duration, frequency	Machine	4 weeks of 30 s stretches/day does not affect extensibility of hamstring muscle group in people with spinal cord injuries.	3
Harvey et al., 2003	16	ROM	Static	Duration, frequency	Machine	4 weeks of 30 s/day stretching no change in ankle mobility of recent injured patients with spinal cord injury.	3
Kilbreath et al., 2012	160	ROM	Staticpassive	Duration, frequency	Loaded	Few impairments reported including swelling following intervention and 6 months post intervention for breast cancer.	3
Lee et al., 2007	64	ROM	Static	Duration, frequency	Loaded	Pectoral stretching no influence on shoulder ROM, strength, arm circumference for women undergoing breast cancer radiotherapy.	3
Maynard et al., 2005	87	ROM	Static	Duration, frequency	Machine	Single session isokinetic and isotonic stretch ankle plantar flexor no clinical significance gait of hemiplegic stroke patients.	3
Moseley et al., 2005	150	ROM	Passive	Duration, frequency	Loaded	No benefit passive and static over exercise alone for treatment plantar flexion post cast immobilization ankle fracture.	3
Putt et al., 2008	14	ROM	PNF	Duration, frequency	Therapist	Hold and relax technique short term benefits for COPD.	3
Turton and Britton, 2005	25	ROM	Static	Duration, frequency	Therapist	Stretching not a workable treatment to prevent contractures.	3
Volpato et al., 2014	14	ROM	Static	Duration, frequency	Therapist	Stabilization exercise or with strengthening proved more effective for improving lumbar pain and flexibility compared to ST stretch.	3
Wang et al., 2008	44	ROM	Static	Duration, frequency	Machine	Ultrasound reliable to assess real-time effects of stretching exercises.	3
Weng et al., 2009	132	ROM	StaticPNF, active	Duration, frequency	Therapist	PNF > Static effectiveness of isokinetic exercise in terms of functional improvement for knee osteoarthritis.
Winters et al., 2004	33	ROM	Activepassive	Duration, frequency	Loaded	Active and Passive ↑ flexibility of tight hip flexor muscles in patients with musculoskeletal issues.	3
Cunha et al., 2008^†^	33	ROM	Static	Intensity D, duration, frequency	Loaded	↑ in pain relief, ROM, quality of life however follow up both groups reported ↑ pain.	2
Hanten et al., 2000^†^	40	ROM	Static	Intensity P, duration, frequency	Machine	Ischemic pressure and sustained stretching effective in ↓ trigger point sensitivity and pain intensity in neck and upper back.	2
Jung et al., 2009	30	ROM	Static	Duration, frequency	Loaded	Standing wall stretch with medial arch subtalar joint ↑ length of Gastrocnemius in subjects with pes planus.	1
Kim et al., 2013	36	ROM	Static	Duration, frequency	Loaded	Compared to the stretching group the ankle muscle strength training group showed statistically sig increases of forward thrust at stroke patients' toe off which positively affected the stroke patients' ability to perform gait.	1
Law et al., 2009	30	ROM	Static	Duration, frequency	Loaded	3 weeks stretching ↑ muscle tolerance not muscle extensibility.	1
Light et al., 1984^†^	11	ROM	StaticPNF	Intensity G, duration, frequency	Loaded	Low-load prolonged stretching (LLPS) is beneficial in the treatment of knee contractures in the immobile nursing home.	1
Ma et al., 2010	43	ROM	Static	Duration, frequency	Loaded	Mini scalpel needling > acupuncture needling or self-neck stretching for Myofascial pain syndrome.	1
Magnusson et al., 1996	10	ROM	StaticPNF	Duration, frequency	Machine	Variable angle protocol demonstrated that PNF stretching altered stretch perception.	1
Renan-Ordine et al., 2011^†^	60	ROM	Static	Intensity D, duration, frequency	LoadedTherapist	Evidence suggests that TrP manual therapies to a self-stretching protocol resulted in superior short-term outcomes vs. self-stretching in treatment of planter heal pain.	2
Trampas et al., 2010^†^	30	ROM	PNF	Intensity D, duration, frequency	Therapist	Myofascial trigger point therapy (MTrP) and modified PNF beneficial for latent MTrP.	2
Triandafilou et al., 2011	15	ROM	Staticpassive	Duration, frequency	Machine	Repetitive Passive ROM > prolonged stretching for improving hand control but not statistically significant.	1
Youdas et al., 2009^†^	22	ROM	Static	Intensity D, duration frequency	Loaded	Active ankle dorsiflexion ROM increased significantly from baseline to week 4, with normal ROM restored with 4 week after acute intervention.	1

MeSH, medical search headings; ROM, range of motion; PNF, proprioceptive neuromuscular facilitation; D, discomfort; P, pain; G, gentle; ↑, increased; ↓, decreased; Q, Quality of Study;

† Indicates study mentioning intensity;

‡ *Indicates high quality study mentioning intensity*.

Of the five remaining lower quality studies referencing intensity (Light et al., 1984; Hanten et al., 2000; Cunha et al., 2008; Trampas et al., 2010; Renan-Ordine et al., 2011), three had participants stretch to discomfort while performing a loaded stretch (Cunha et al., 2008; Renan-Ordine et al., 2011) or with use of a therapist (Trampas et al., 2010). Of the remaining two studies Hanten et al. had patients stretch to pain with use of a machine (Hanten et al., 2000) and Light et al. used a loaded stretch with a gentle stretch intensity (Light et al., 1984). Interestingly, amongst the seven studies referencing intensity, two studies observed the response of participants to global postural re-education, a technique that simultaneously stretches all the muscles in either a posterior or anterior muscle chain. These studies contradicted each other, with the high quality study indicating an increase in pain relief (Maluf et al., 2010), with the other study reporting an increase in pain post-intervention (Cunha et al., 2008). This contradiction exemplifies the current state of knowledge regarding stretch intensity and position and further suggests the need for higher quality research studies. It may be the need to standardize methodologies and data collection in order to ensure better outcomes. A contradiction was also observed between two low quality studies concerned with trigger points (TrP) (Trampas et al., 2010; Renan-Ordine et al., 2011). Renan-Ordine et al. suggested that trigger point (TrP) manual therapies were superior over use of self-stretching (Renan-Ordine et al., 2011), with Trampas et al. indicating that stretching was more beneficial for latent myofascial TrPs (Trampas et al., 2010). In addition, another study that looked at a combination of a gentle intensity with a loaded stretch, reported a benefit in the treatment of knee contractures (Light et al., 1984). It is interesting to note that a study combining stretch to discomfort through a therapist, and a study combining a loaded stretch with gentle intensity both showed a benefit. The use of a therapist may well have increased stability and the ability of the participants to relax, which yielded the same effect to that seen with use of a gentle loaded stretch. This observation also applies to the last study, which reported a decrease in TrP sensitivity and pain intensity in the neck following stretching to an intensity of pain via use of a machine (Hanten et al., 2000); here, the machine provided a stable controlled environment, possibly allowing participants to relax during the intervention.

Out of the 29 studies 15 studies which did not mention intensity, rated a three out of five using the quality criteria scores (Harvey et al., 2000, 2003; Winters et al., 2004; Maynard et al., 2005; Moseley et al., 2005; Turton and Britton, 2005; Gustafsson and McKenna, 2006; Häkkinen et al., 2007; Lee et al., 2007; Putt et al., 2008; Wang et al., 2008; Weng et al., 2009; Kilbreath et al., 2012; Albayrak et al., 2014; Volpato et al., 2014). Of these high quality studies, six studies revealed no benefit regarding spinal cord and contracture issues with use of static stretching (Harvey et al., 2000, 2003; Maynard et al., 2005; Turton and Britton, 2005; Gustafsson and McKenna, 2006; Horsley et al., 2007). Four studies on spinal cord injuries used a machine to stabilize stretch position (Harvey et al., 2000, 2003; Maynard et al., 2005; Gustafsson and McKenna, 2006) and the one study on contractures used a therapist to provide the stretch (Turton and Britton, 2005). It has been observed that stretching does not affect CNS injuries and disorders, but alters PNS function. This view is supported by five studies which indicate that static stretching was beneficial for treating neck pain (Häkkinen et al., 2007), a positive morphologic change of the iliotibial band (Wang et al., 2008), knee osteoarthritis (Weng et al., 2009) and an increase of hip flexor ROM (Winters et al., 2004) as well tight hip flexors (Winters et al., 2004). Participants were stretched via a machine (Wang et al., 2008), a therapist (Häkkinen et al., 2007; Putt et al., 2008; Weng et al., 2009), or performed a loaded stretch (Winters et al., 2004). The study by Albayrak also supported the view of the influence of stretching on the PNS observing that an increase in mobility improved both the depression level and health status of the patients (Albayrak et al., 2014).

### Elderly population

Twelve studies with designated control groups, reported the benefits of stretching in the elderly. Improvements were seen in various outcomes, including gait length and speed as well as flexibility and mobility (Feland et al., 2001; Kerrigan et al., 2003; Gadjosik et al., 2005; Zakas et al., 2006; Christiansen, 2008; Batista et al., 2009; Cristopoliski et al., 2009; Stanziano et al., 2009; González-Ravé et al., 2012; Locks et al., 2012; Watt et al., 2011a,b) (Table 3). All the articles reported duration and frequency, with five referring to stretch intensity of which two were of high quality (Zakas et al., 2006; Cristopoliski et al., 2009), with three being of low quality (Feland et al., 2001; Batista et al., 2009; González-Ravé et al., 2012).

**Table 3:** **Elderly population**.

**Article**	***n***	**Methodology**				**Main outcomes**	**Q**
		**MeSH term**	**Type of stretch**	**Elements of stretching**	**Stretch position**		
Christiansen, 2008	40	ROM	Static	Duration, frequency	Supported	Intervention group ↑ hip and knee and ankle motion and gait speed.	4
Kerrigan et al., 2003	96	ROM	Static	Duration, frequency	Supported	↑ static peak hip extension, comfortable and fast walking speed, dynamic hip extension, peak ankle plantar flexion, and ankle power generation.	4
Watt et al., 2011a	82	ROM	Static	Duration, frequency	Supported	10 week flexibility ↑ stride length, peak hip extension during walking.	4
Watt et al., 2011b	74	ROM	Static	Duration, frequency	Supported	↑ walking speed, stride length, passive hip extension ROM.	4
Cristopoliski et al., 2009^‡^	20	ROM	Static	Intensity D, duration, frequency	Therapist	Experimental group ↑ step length, higher velocity ↓ double support time after training and ↑ anterior and lateral pelvis tilt and greater rotation.	3
Locks et al., 2012	45	ROM	StaticPNF	Duration, frequency	Therapist	6 weeks stretching/resistive training ↑ functional status of older people, Diastolic Blood Pressure ↓, detraining ↑ SBP when resistive exercise used alone.	3
Zakas et al., 2006^‡^	22	ROM	Static	Intensity G, duration, frequency	Supported	results indicate immediate changes in flexibility via acute stretching exercise in sedentary elderly women when muscle undergo static elongation, irrespective of performance of warming up exercises.	3
Batista et al., 2009^†^	12	ROM	Active	Intensity MSNP, duration, frequency	Supported	Active stretching program was effective in increasing the flexibility of knee flexors, extensor and flexor torque, and functional mobility in older women.	1
Feland et al., 2001^†^	62	ROM	Static	Intensity D, duration, frequency	Therapist	Longer hold times 60 s > 30 s > 15 s greater rate of gains and more sustained ↑ ROM in elderly.	2
Gadjosik et al., 2005	19	ROM	Static	Duration, frequency	Machine	Stretching group ↑ max dorsiflexion Passive ROM, ↑ passive resistive force ↑ absorbed and retained passive elastic energy. ↓ time agility course and 10-m walk.	2
González-Ravé et al., 2012^†^	54	ROM	StaticPNF, passive	Intensity D, duration, frequency	Therapist	Physically active older people ↑ ROM in response to stretching techniques similar for both passive and PNF techniques.	1
Stanziano et al., 2009	17	ROM	PNF	Duration, frequency	Machine	8 week flexibility ↓ age related losses ROM ↑ functional performance.	2

MeSH, medical search headings; ROM, range of motion; PNF, proprioceptive neuromuscular facilitation; ↑, increased; ↓, decreased; Q, quality of study;

† Indicates study mentioning intensity;

‡ *Indicates high quality study mentioning intensity*.

The two high quality studies had the elderly participants perform a supported stretch (Zakas et al., 2006) or were stretched by a therapist using a static stretch (Cristopoliski et al., 2009). One study observed an improvement in flexibility (Zakas et al., 2006), with the other seeing an increase in step length resulting in an increase in higher velocity during walking (Cristopoliski et al., 2009). Five studies which rated a high quality but did not mention stretch intensity used static stretching (Kerrigan et al., 2003; Christiansen, 2008; Watt et al., 2011a,b), or in combination with a PNF stretch (Locks et al., 2012). All participants performed a supported and stable stretch, which resulted in an increase in gait, walking speed, stride length, ROM for hip extension, and plantar flexion.

Of the remaining five studies which were low quality, three mentioned intensity, with one study having participants perform a static stretch with use of a therapist (Feland et al., 2001), another with use of a therapist exposed the elderly to a static stretch in combination with PNF and passive (González-Ravé et al., 2012), with the last study having the elderly perform an active supported stretch (Batista et al., 2009). All three observed that stretching was beneficial with regard to increasing flexibility of knee flexors, extensors (Batista et al., 2009), as well as increasing ROM of the hamstring, hip, and shoulder (Feland et al., 2001; González-Ravé et al., 2012). The other studies used machines to induce stretching, and found an increase in dorsiflexion and passive resistance force, and a decrease in age-related losses of ROM (Gadjosik et al., 2005; Stanziano et al., 2009). It is interesting to note that with the elderly population all the studies had the participants perform a supported stretch, or made use of a therapist or a machine. None of the participants performed loaded stretches.

### General population

A total of 87 articles were included in the general population, with all articles referencing duration and frequency, and 55 mentioning intensity (Table 4). Of these 55 articles 15 were of high quality and 40 of low quality. Within the high quality studies, one study had participants stretch to pain during a loaded stretch, and reported that static stretching was more likely compared to ballistic to cause DOMS (Smith et al., 1993). Six studies had participants stretch to discomfort, with five of these having participants perform a loaded stretch (McNair and Stanley, 1996; Curry et al., 2009; Rancour et al., 2009; Cipriani et al., 2012; Wicke et al., 2014). The last study made use of a machine to generate stress on the muscle tendon unit during the stretch (Medeiros et al., 1977). The study by Medeiros et al. indicated both isometric and passive stretching produce similar results on hip joint ROM (Medeiros et al., 1977). McNair et al. observed that a combination of a loaded stretch with discomfort was more effective for increasing dorsiflexion compared to jogging (McNair and Stanley, 1996). The study by Wicke et al. (2014) suggested that self-PNF can be used in place of static stretching to increase ROM about a joint. The study by Cipriani et al. (2012) indicated no sex differences in terms of stretch response, with Curry et al. (2009) observing that dynamic flexibility enhanced performance on power outcomes greater than static stretching. The study by Rancour et al. (2009) suggested that intermittent stretch training (i.e., 2 or 3 times per week) was sufficient to maintain ROM gains acquired from a static stretching programme.

**Table 4:** **General population**.

**Article**	***n***	**Methodology**				**Main outcomes**	**Q**
		**MeSH term**	**Type of stretch**	**Elements of stretching**	**Stretch position**		
Smith et al., 1993^‡^	20	DOMS	Staticballistic	Intensity P, duration, frequency	Loaded	Similar bouts of static and ballistic stretching induce significant ↑ in DOMS and Creatine Kinase to subjects unaccustomed to such exercise.	3
Apostolopoulos et al., 2015a^‡^	12	ROM	Static	Intensity P, duration, frequency	Therapist	It was observed that intense stretching may lead to an acute inflammatory response supported by a significant increase in hsCRP.	3
Apostolopoulos et al., 2015b^‡^	11	ROM	Static	Intensity G, P, duration, frequency	Machine	The optimal intensity for not causing inflammation was observed to be between 30 and 60% of an individual's max ROM.	3
Borman et al., 2011^‡^	36	ROM	Static	Intensity MSNP, duration, frequency	Loaded	No change in lumbar ROM or curvature even though ↑ hamstring length.	3
Cipriani et al., 2012^‡^	53	ROM	Static	Intensity D, duration, frequency	Loaded	Stretching equally effective, whether performed daily or 3×/week. provided individuals stretch 2×/day. No sex difference in terms of stretch response.	3
Clark et al., 1999^‡^	60	ROM	StaticPNF	Intensity MSNP, duration, frequency	Therapist	Sagittal hold relax and passive prone ↑ straight-leg ROM with sagittal hold relax > passive prone.	3
Curry et al., 2009^‡^	24	ROM	Staticdynamic	Intensity D, duration, frequency	Loaded	Dynamic flexibility greater applicability vs. static regards enhancing performance on power outcomes.	3
de Weijer et al., 2003^‡^	56	ROM	Static	Intensity MSNP, duration, frequency	Loaded	Significant ↑ hamstring length maintained up to 24 h using static. Warm Up prior to static no significant ↑ hamstring length.	3
McNair and Stanley, 1996^‡^	24	ROM	Static	Intensity D, duration, frequency	Loaded	↑ dorsiflexion ROM at ankle combination of jogging and stretching and stretching alone greater than jogging.	3
Medeiros et al., 1977^‡^	30	ROM	PNFpassive	Intensity D, duration, frequency	Machine	Comparisons between two treatment groups indicated isometric contraction and passive stretch procedures significant and similar effects.	3
Muir et al., 1999^‡^	20	ROM	Static	Intensity MSNP, duration, frequency	Loaded	Static calf-stretching short duration no ↓ passive resistance of connective tissue within surrounding muscle and joint structures of ankles (healthy).	3
O'sullivan et al., 2009^‡^	36	ROM	Staticdynamic	Intensity G, duration, frequency	Loaded	Warm-up and Static significant ↑ hamstring flexibility whereas dynamic did not.	3
Rancour et al., 2009^‡^	35	ROM	Static	Intensity D, duration, frequency	Loaded	Intermittent stretching (i.e., 2 or 3 days/week) is sufficient to maintain ROM gains acquired from prior static program.	3
Wicke et al., 2014^‡^	19	ROM	StaticPNF	Intensity D, duration, frequency	Loaded	The results suggest that self PNF can be used in place of PNF stretching giving control of the stretching to the individual.	3
Youdas et al., 2003^‡^	101	ROM	Static	Intensity MSNP, duration, frequency	Loaded	6-week static program once/day up to 2 min. not sufficient for ↑ active dorsiflexion ROM.	3
Bandy et al., 1997	93	ROM	Static	Duration, frequency	Loaded	No ↑ flexibility when duration ↑ from 30 to 60 s or frequency from 1 to 3 times/day.	3
Ben and Harvey, 2010	60	ROM	Static	Duration, frequency	Machine	No ↑ muscle extensibility with 6 weeks of 30 min daily stretch however ↑ stretch tolerance.	3
Chadchavalpanichaya and Srisawasdi, 2010	80	ROM	Static	Duration, frequency	Loaded	Use of calf box ↑ compliance ↓ calf muscle tightness and complications vs. conventional method.	3
Gribble et al., 1999	42	ROM	StaticPNF	Duration, frequency	Machine	Static and hold-relax stretching equally effective in ↑ hamstring ROM. Both goniometer and FlexAbility LE1000 reliable measuring hip-flexion ROM.	3
Ross, 2007	13	ROM	Static	Duration, frequency	Loaded	15 day stretching program with purposeful delay b/n stretching and performance ↓ effect caused by acute stretching.	3
Rowlands et al., 2003	43	ROM	PNF	Duration, frequency	Therapist	A longer contraction time leads to greater ↑ in flexibility.	3
Sainz de Baranda and Ayala, 2010	173	ROM	Staticactive	Duration, frequency	Loaded	Recommendations of current ACSM flexibility training effective for ↑ hip flexion ROM in recreationally active young adults.	3
Starring et al., 1988	|43	ROM	Passive	Duration, frequency	Machine	Cycling passive > sustained passive stretch in ROM gains.	3
Rodenburg et al., 1994^†^	50	INFL	Static	Intensity D, duration, frequency	Therapist	Combination warm-up, stretching and massage ↓ some negative effects results inconsistent.	2
Almasi et al., 2014	16	DOMS	PNF	Duration, frequency	Therapist	Combination treatment (PNF and therapeutic massage) were effective on maintenance isometric strength and decreased pain DOMS and pain intensity rate.	1
Johansson et al., 1999^†^	10	DOMS	Static	Intensity MSNP, duration, frequency	Loaded	Pre-exercise stretching no preventive effect on muscle soreness, tenderness and force loss following eccentric exercise.	1
Krityakiarana et al., 2014	55	DOMS	Dynamic	Duration, frequency	Loaded	Results non-significant that combined treatment may be contraindicated in prevention of DOMS and ice bag or DS might be best choice of treatment.	1
LaRoche, 2006^†^	29	DOMS	Staticballistic	Intensity MSNP, duration, frequency	Loaded	Static and Ballistic ↑ ROM enhanced tolerance vs. changes in muscle elasticity.	1
McGrath et al., 2014^†^	57	DOMS	Static PNF	Intensity D, duration, frequency	LoadedTherapist	Similar to SS results indicate that post-exercise PNF stretching does not prevent DOMS.	2
Wessel and Wan, 1994^†^	10	DOMS	Static	Intensity D, duration, frequency	Loaded	Stretching protocol pre- and post-eccentric exercise no reduction of DOMS.	2
Aguilar et al., 2012^†^	45	ROM	Staticdynamic	Intensity D, duration, frequency	Loaded	Dynamic > static warm-up for pre-activity acutely ↑ses strength production of quadriceps and hamstring flexibility, with ↓ injury risk.	1
Aquino et al., 2010^†^	45	ROM	Static	Intensity MSNP, duration, frequency	Loaded	Stretching no change in torque-angle curve and flexibility therefore ↑ tolerance.	2
Ayala et al., 2012	50	ROM	Active	Duration, frequency	Loaded	12 week active stretching program ↑ hip flexion passive ROM when performed 3 days/week. with daily dose of 180 s (6 × 30 s) in both normal and limited hamstring flexibility.	1
Ayala and de Baranda Andújar, 2010	150	ROM	Active	Duration, frequency	Loaded	No sig diff b/n 3 treatment groups. 12 × 15 s 6 × 30 s, 4 × 45 s, equally effective for ↑ hamstring length.	1
Azevedo et al., 2011^†^	60	ROM	PNF	Intensity MSNP, duration, frequency	Therapist	ROM gain with contract-relax PNF same whether target muscle or uninvolved muscle is contracted.	2
Bandy et al., 1998^†^	48	ROM	Staticdynamic	Intensity G, duration, frequency	Loaded	30 s static stretch ↑ ROM > dynamic ROM.	1
Bannerman et al., 1996	44	ROM	Staticballistic	Duration, frequency	Loaded	15 s stretch performed twice weekly produces a significant ↑ soleus muscle length regardless of stretch type.	1
Beedle et al., 2007	30	ROM	Static	Duration, frequency	Loaded	Stretching pre- or post-workout no difference on flexibility ↑.	1
Blazevich et al., 2014^†^	22	ROM	Static	Intensity P, duration, frequency	Loaded	Increases in end ROM were underpinned by increases in max tolerable passive joint moment (stretch tolerance) rather than change in volitional muscle activation or motorneuron pool excitability.	2
Bonnar et al., 2004	60	ROM	PNF	Duration, frequency	Therapist	All three-hold time conditions of PNF ↑ ROM in hip joint flexibility.	1
Cabido et al., 2014^†^	23	ROM	Static	Intensity D, duration, frequency	Machine	Greater increase in ROM may be explained by both changes in biomechanical properties of muscle tendon unit and stretch tolerance.	1
Chan et al., 2001^†^	45	ROM	Static	Intensity D, duration, frequency	Loaded	Both protocols 4 or 8 week were effective in ↑ hamstring flexibility.	2
Chen et al., 2013^†^	9	ROM	StaticPNF	Intensity D, duration, frequency	LoadedTherapist	Stretching protocols improve hamstring flexibility immediately, after exercise hamstrings peak torque diminished for SS + PNF but not for SS+ Taping. Therefore, SS + Taping can prevent –ve results from exercise.	2
Cornelius et al., 1992	120	ROM	PNFpassive	Duration, frequency	Therapist	*Post-hoc* analyses 3 modified PNF techniques ↑ ROM vs. passive stretch technique. Cold application no influence stretching techniques.	1
Cornelius et al., 1995^†^	60	ROM	PNF	Intensity D, duration, frequency	Therapist	All PNF treatments ↑ ROM. Trials 1 and 2 PNF ↑ ROM with no ↑ Systolic BP 3rd trial ↑ SBP.	2
Decoster et al., 2004^†^	29	ROM	Static	Intensity MSNP, duration, frequency	Loaded	Standing and supine hamstring stretches both effective ↑ flexibility.	2
deCarvalho et al., 2011	44	ROM	Static	Duration, frequency	Therapist	Acupuncture application in acupoint and no-acupoints before exercises of SS can generate an acute significant increase on hip ROM.	1
Fantini et al., 2006^†^	30	ROM	Static	Intensity D, duration, frequency	Therapist	Results showed an increase in ground contact time, knee and ankle flexion and knee max angular velocity during eccentric and concentric phases. However, these changes in the movement technique have not affected the performance.	1
Fasen et al., 2009	100	ROM	PNFpassive	Duration, frequency	Loaded	↑ hamstring flexibility greatest for straight leg raise passive stretch. PNF 90/90 active stretch > 90/90 passive with ↑ knee ROM.	1
Freitas and Mil-Homens, 2015^†^	10	ROM	Static	Intensity D, duration, frequency	Loaded	8 week high-intensity stretching programme was observed to efficiently increase the biceps femoris fascicle length as well as the knee extension max ROM.	1
Ghaffarinejad et al., 2007	39	ROM	Static	Duration, frequency	Machine	Accuracy of knee joint position sensation in 45° flexion improved subsequent to static stretch of quads, hamstrings, adductors.	2
Halbertsma and Goeken, 1994^†^	14	ROM	Static	Intensity P, duration, frequency	Loaded	Stretching exercises do not make short hamstrings longer or less stiff, only influence stretch tolerance.	1
Hayes et al., 2012^†^	40	ROM	Static	Intensity D, duration, frequency	Loaded	Mechanical and/or stretch tolerance has a greater influence on ↑ ankle-dorsiflexion passive ROM after long-term stretching protocol (6 weeks and 30 sessions).	1
Herda et al., 2012^†^	11	ROM	Static	Intensity D, duration, frequency	Machine	Constant-torque stretching may be more appropriate than stretch held at a constant muscle length ↓ musculo-tendinous stiffness.	1
Johanson et al., 2008	33	ROM	Static	Duration, frequency	Loaded	Subtalar joint position no influence on gains in ankle dorsiflexion ROM post gastrocnemius stretching program.	2
Johanson et al., 2009^†^	16	ROM	Static	Intensity MSNP, duration, frequency	Loaded	Stretching did not alter joint angles or gastrocnemius muscle activity in early to midstance phase of gait.	2
Johanson et al., 2006^†^	19	ROM	Static	Intensity MSNP, duration, frequency	Loaded	Gastrocnemius stretch 2×/day for 3 weeks ↑ passive ankle dorsiflexion but no change in ankle dorsiflexion or time-to-heel-off during stance phase of gait.	1
Kasser et al., 2009^†^	27	ROM	Static	Intensity G, duration, frequency	Loaded	Appropriate for patients lacking ankle dorsiflexion to strengthen the muscle in the anterior compartment of the leg with a stretching program.	2
Klinge et al., 1997	22	ROM	Static	Duration, frequency	Machine	↑ isometric strength is accompanied by changes in material properties of muscle are unaffected by flexibility exercises.	1
Kokkonen et al., 1998^†^	30	ROM	Staticactive	Intensity P, duration, frequency	Loaded	Maximal knee-flexion and extension 1RMs can be ↓ by acute stretching. intense static stretching of prime movers should be avoided for max strength output.	1
Konrad and Tilp, 2014^†^	49	ROM	Static	Intensity D, duration, frequency	Loaded	Increased ROM could not be explained by the structural changes in the MTU and was likely due to increased stretch tolerance possibly due to adaptation of nociceptive nerve endings.	1
Mahieu et al., 2009^†^	62	ROM	PNF	Intensity D, duration, frequency	Loaded	PNF stretching ↑ ankle dorsiflexion ↑ stretch tolerance.	2
Mahieu et al., 2007^†^	81	ROM	Staticballistic	Intensity D, duration, frequency	Loaded	Static and ballistic stretching different effects on passive resistive torque and tendon stiffness both should be considered for training and rehabilitation.	2
McCarthy et al., 1997	40	ROM	PNF	Duration, frequency	Loaded	Stretching exercises ↑ cervical ROM short term. For continued effect need to continue stretching regime.	1
McClure et al., 2007^†^	54	ROM	Static	Intensity D, duration, frequency	Loaded	Sleeper stretch > Cross body stretch for ↑ ROM.	2
McNair et al., 2000	24	ROM	Staticactive	Duration, frequency	Machine	↓ stiffness continuous > holds. If peak tension aim holds > continuous.	1
Meroni et al., 2010^†^	65	ROM	Activepassive	Intensity D, duration, frequency	Loaded	Active > passive in active knee extension ROM test and maintained completely 4 weeks post training.	2
Minshull et al., 2014	18	ROM	Passive PNF	Duration, frequency	Therapist	PNF was efficacious in flexibility conditioning suggesting that this be used over passive to help preserved dynamic joint stability.	1
Mizuno et al., 2012^†^	11	ROM	Static	Intensity D, duration, frequency	Machine	Static stretch 5 s significant ↑ ROM over 30 s but significant ↓ in stiffness of muscle tendon unit returning to baseline within 5–10′.	2
de Oliveira et al., 2012^†^	15	ROM	Static	Intensity D, duration, frequency	Loaded	Explosive muscular actions of a very short duration (100 ms) seem less affected by active SS when compared with actions using maximal muscle strength.	2
Moreside and McGill, 2012	24	ROM	Staticballistic	Duration, frequency	Loaded	Stretches aimed at myofascial components of upper body and hip joint ↑ hip ROM.	2
Muanjai and Namsawang, 2015^†^	45	ROM	Static	Intensity D, duration, frequency	Loaded	SS or cold water immersion alone is are more effective than combined SS and cold immersion with regard to DOMS.	2
Murphy et al., 2010^†^	11	ROM	Static	Intensity P, duration, frequency	Loaded	The aerobic-static-aerobic (ASA) method provide ROM improvements for 30 s with no impairment in performance vs. traditional warm-up.	1
Nakamura et al., 2012^†^	18	ROM	Static	Intensity D, duration, frequency	Loaded	4-week static training changes flexibility of overall muscle tendon unit with no changes in muscle fascicle length.	1
O'Hora et al., 2011^†^	45	ROM	StaticPNF	Intensity D, duration, frequency	Therapist	Hamstring (agonist) contract PNF > Static in a single stretch session.	2
Place et al., 2012	12	ROM	PNF	Duration, frequency	Loaded	Self-administered PNF stretching of the quadriceps with short 5 s stretches not recommended before sports where flexibility is mandatory for performance.	1
Puentedura et al., 2011	13	ROM	StaticPNF	Duration, frequency	Therapist	No sig difference between Hold-Relax PNF and Static for ↑ hamstring length.	2
Rees et al., 2007^†^	20	ROM	PNF	Intensity MSNP, duration, frequency	Machine	PNF useful modality for ↑ joint ROM and strength.	1
Ryan et al., 2008b^†^	12	ROM	Passive	Intensity D, duration, frequency	Machine	Practical durations of passive stretching resulted in significant decreases in MTS; however these changes return to baseline levels within 10–20 min.	1
Ryan et al., 2008a^†^	13	ROM	Passive	Intensity D, duration, frequency	Machine	Durations (2, 4, 8) min plantarflexors no ↓ isometric peak torque vs. control group. Some ↑ ROM, thereby questioning overall influence of passive stretch on performance.	1
Davis et al., 2005^†^	19	ROM	StaticPNF, active	Intensity MSNP, duration, frequency	Therapist	Static 1 rep for 30 s 3 days/week > active self-stretching and PNF for ↑ hamstring length.	1
Torres et al., 2007	30	ROM	Static	Duration, frequency	Therapist	Stretching program conducted after exhaustive eccentric exercise alleviated reductions in ROM induced exercise.	1
Webright et al., 1997	40	ROM	Staticactive	Duration, frequency	Loaded	6 weeks non-ballistic, repetitive active knee extension (30 reps 2×/day) neural slump sitting position ↑ hamstring flexibility no difference with Static (30″, 2×/day).	1
Wiemann and Hahn, 1997	60	ROM	Staticballistic	Duration, frequency	Loaded	Static and ballistic ↑ ROM and ↑ tolerance to higher stretching strain.	1
Winke et al., 2010^†^	29	ROM	Static	Intensity D, duration, frequency	Machine	Moderate static no impact on performance ↓ in concentric or eccentric torque output at slow or fast contraction velocities.	1
Youdas et al., 2010	35	ROM	PNF	Duration, frequency	Loaded	10-s modified hold-relax PNF procedure produced an 11° gain in knee extension angle within a single stretch session.	2
Yuktasir and Kaya, 2007	28	ROM	StaticPNF	Duration, frequency	Therapist	Static and PNF stretching techniques improve ROM, but neither stretching exercises had any statistically significant effect on the drop jump scores.	1

MeSH, medical search headings; ROM, range of motion; PNF, proprioceptive neuromuscular facilitation; D, discomfort; P, pain; MSNP, maximum stretch no pain; ↑, increased; ↓, decreased; Q, quality of study;

† Indicates study mentioning intensity;

‡ *Indicates high quality study mentioning intensity*.

Of the remaining eight high quality studies, four referred to MSNP during a loaded stretch position (Muir et al., 1999; de Weijer et al., 2003; Youdas et al., 2009; Borman et al., 2011), one observed the use of a therapist during a MSNP stretch (Clark et al., 1999), with the last observing the results of a gentle loaded stretch (O'sullivan et al., 2009). Overall, it is interesting to note that articles referring to stretches performed with MSNP and gentle indicated a benefit within the participants compared to the stretches utilizing pain and discomfort during a loaded stretch. Finally, two studies referred to stretch intensity, with use of a therapist and a machine (Apostolopoulos et al., 2015a,b). In the first study participants were stretched to pain with use of a therapist concluding that an intense stretch can cause inflammation (Apostolopoulos et al., 2015a). Similarly, the second study, which used a machine to stretch participants at various stretch intensities concluded that a very intense stretch caused inflammation (Apostolopoulos et al., 2015b).

Regarding the 40 low quality studies, 25 had participants stretch to discomfort of which 15 in combination with a loaded stretch (Wessel and Wan, 1994; Chan et al., 2001; Mahieu et al., 2007; McClure et al., 2007; Mahieu et al., 2009; Meroni et al., 2010; Aguilar et al., 2012; de Oliveira et al., 2012; Hayes et al., 2012; Morais de Oliveira et al., 2012; Nakamura et al., 2012; Chen et al., 2013; Konrad and Tilp, 2014; McGrath et al., 2014; Freitas and Mil-Homens, 2015; Muanjai and Namsawang, 2015), four used therapists (Rodenburg et al., 1994; Cornelius et al., 1995; Fantini et al., 2006; O'Hora et al., 2011), while the remaining six made use of machines (Ryan et al., 2008a,b; Winke et al., 2010; Herda et al., 2012; Mizuno et al., 2012; Cabido et al., 2014). In turn, of the 25 articles four suggested that the changes observed with stretching were due to stretch tolerance rather than mechanical (Mahieu et al., 2009; Hayes et al., 2012; Cabido et al., 2014; Konrad and Tilp, 2014). In contrast four articles observed changes in the musculoskeletal system in response to stretching (Herda et al., 2012; Mizuno et al., 2012; Nakamura et al., 2012; Freitas and Mil-Homens, 2015).

Of the 15 remaining articles, nine referred to MSNP, with six referencing a loaded stretch (Johansson et al., 1999; Decoster et al., 2004; Johanson et al., 2006, 2009; LaRoche, 2006; Aquino et al., 2010), two with a therapist (Davis et al., 2005; Azevedo et al., 2011), and the last one had participants perform the stretch with use of a machine (Rees et al., 2007). Similar to the articles that referred to discomfort, two indicated that stretch tolerance was the reason for the observed increase in ROM (LaRoche, 2006; Aquino et al., 2010).

Four articles had participants stretch to pain, three performing a loaded stretch (Kokkonen et al., 1998; Murphy et al., 2010; Blazevich et al., 2012) and one with a therapist (Halbertsma and Goeken, 1994). Two studies suggested that the increase in ROM was due to stretch tolerance (Halbertsma and Goeken, 1994; Blazevich et al., 2014). The last two low quality studies referred to gentle loaded stretches (Bandy et al., 1998; Kasser et al., 2009).

Fifty-eight studies referenced static stretching by itself or in combination with other stretching techniques concerned with ROM (Table 4). Four studies referred to static stretching or in combination of with regard to DOMS (Wessel and Wan, 1994; Johansson et al., 1999; LaRoche, 2006; McGrath et al., 2014). It is interesting to note that all four had participants perform a loaded stretch.

When comparing articles that referenced loaded stretches to either therapists, machines when referring to discomfort, pain, MSNP, and gentle, it was observed that articles using therapists, and machines indicated an improvement in ROM overall (Rodenburg et al., 1994; Cornelius et al., 1995; Davis et al., 2005; Fantini et al., 2006; Rees et al., 2007; Ryan et al., 2008a,b; Herda et al., 2012; Mizuno et al., 2012). In contrast, when a stretch using a therapist was performed to pain this did not improve the ROM of the hamstring muscles (Halbertsma and Goeken, 1994). Further investigation is needed to determine the reason for the observed influence of the therapist and machine with use of comparing MSNP vs. pain.

Overall the 40 low quality studies were inconclusive as to whether stretching was beneficial. This discrepancy may be due to the influence of discomfort or pain with use of a loaded stretch, vs. use of a machine or therapist were there is possibly more control and support during the execution of the stretch. More research is needed to be performed to determine if this is the case or not.

It is worthwhile to note that 10 studies lacked a designated control group (Cornelius et al., 1995; Kokkonen et al., 1998; McNair et al., 2000; Beedle et al., 2007; Ghaffarinejad et al., 2007; Torres et al., 2007; Meroni et al., 2010; Murphy et al., 2010; Winke et al., 2010; Cabido et al., 2014), with only four studies referencing all three stretching parameters (intensity, duration, and frequency). Of these four studies, three used a loaded stretch position (Kokkonen et al., 1998; Meroni et al., 2010; Murphy et al., 2010) with two referencing pain, and another last referring to discomfort. Unfortunately, the lack of a control group makes these studies inconclusive, reinforcing the need to design and conduct higher quality studies in order to properly observe and determine the importance of stretch intensity and position.

## Discussion

The aim of this review was to examine the relevance of stretch intensity and position in different populations, and to investigate the potential relationship of the two in terms of inflammation, DOMS and ROM. A total of 152 articles were identified for this literature review. The majority of the studies in each of the four populations were of low quality based on the “quality of study” criteria selected for this review. Based on the criteria used there is a need for higher quality material regarding these important exercise and training elements and their influence on athletic performance as well as rehabilitation.

A common theme in the four populations is that the associated studies refer mainly to duration and frequency, with only a few referencing intensity. The likely reason for this is that duration and frequency are easier to manipulate and quantify (Feland et al., 2001). Concerning stretch intensity and position, discrepancies were prevalent within the four groups. In the athletic group, all the studies dealt with ROM, with only 12 mentioning intensity (Magnusson et al., 1998; Roberts and Wilson, 1999; Hayes and Walker, 2007; Allison et al., 2008; Bazett-Jones et al., 2008; Caplan et al., 2009; Favero et al., 2009; Tsolakis et al., 2010; Silveira et al., 2011; Maenhout et al., 2012; Morrin and Redding, 2013; Wyon et al., 2013). It is noticeable that five (Hayes and Walker, 2007; Allison et al., 2008; Bazett-Jones et al., 2008; Favero et al., 2009; Silveira et al., 2011) out of the seven studies (Roberts and Wilson, 1999; Hayes and Walker, 2007; Allison et al., 2008; Bazett-Jones et al., 2008; Caplan et al., 2009; Favero et al., 2009; Silveira et al., 2011) which combined a loaded stretch with an intensity of discomfort and pain observed no improvement. The one study that referred to a supported stretch while participants were performing a stretch of either a gentle or discomfort intensity, observed that the participants performing a gentle supported stretch had the greatest gains in both active and passive ROM (Wyon et al., 2013). This begs the question as to whether a loaded stretch may influence stretch intensity. However, these studies that referred to discomfort and pain during a loaded stretch (Allison et al., 2008; Bazett-Jones et al., 2008; Caplan et al., 2009; Favero et al., 2009) were of low quality, thus preventing definite conclusions to be drawn based on the criteria of the literature review that looked at higher quality studies which mentioned stretch intensity.

In line with the athlete group, studies comprising the clinical equivalent were primarily focused on ROM. However, greater dependency was placed on the use of therapists and machines to achieve optimal stretch position. Regarding intensity, two studies examining global posture re-education referred to intensity during a loaded stretch position (Cunha et al., 2008; Maluf et al., 2010). Unfortunately, contradictory results were reported with one (Cunha et al., 2008) (low quality study) indicating an increase in pain at follow up, and the other (Maluf et al., 2010) whereas the latter (high quality study) did not report this finding. In contrast, two low quality studies making use of a supported stretch position revealed beneficial effects (Hanten et al., 2000; Trampas et al., 2010). It seems likely that this supported position may allow for better stability assuring a better control and application of stretch intensity (Wyon et al., 2009). It should be noted, that though a large number of the studies where of high quality, the focus was whether these studies mentioned intensity and at what level was the intensity of the stretch (i.e., discomfort, pain, gentle).

The elderly population was also concerned with the influence of stretching exercises on ROM. Unlike the other populations, the elderly group did not have participants perform a loaded stretch, possibly anxious about loading an aged muscle. Most of the studies were of high quality. In general, the observed benefits in this population were related to the participants performing a supported stretch or being stretched by a therapist in a supported position (i.e., lying on a plinth). With the muscles and connective tissue in a stable environment, greater control can be imparted on the magnitude of the intensity. This is important since several age-related musculoskeletal and physiological changes such as muscle atrophy, reduced capacity for healing, and loss of strength and elasticity has been associated with the elderly (Feland et al., 2001).

In line with the other groups reported herein, most of the studies in the general population focused on ROM. Referring to a loaded stretch in conjunction with intensity (i.e., discomfort, pain, and MSNP), no definitive trend emerged as to the benefit of this combination. However, studies mentioning stretch intensity during a supported stretch position (machine and/or therapist) did reveal a benefit (Rees et al., 2007; Winke et al., 2010). With use of therapists to stretch participants, greater support of the musculo-tendinous structure could account for the decrease in intensity of the stretch during stretching.

It is noteworthy that although all 152 articles considered duration and frequency, only 79 (51.33%) referred to intensity. Of these 79 articles, only 22 (27.84%) (Medeiros et al., 1977; Smith et al., 1993; McNair and Stanley, 1996; Clark et al., 1999; Muir et al., 1999; de Weijer et al., 2003; Youdas et al., 2003; Zakas et al., 2006; Hayes and Walker, 2007; Horsley et al., 2007; Cristopoliski et al., 2009; Curry et al., 2009; O'sullivan et al., 2009; Rancour et al., 2009; Maluf et al., 2010; Borman et al., 2011; Silveira et al., 2011; Cipriani et al., 2012; Wyon et al., 2013; Wicke et al., 2014; Apostolopoulos et al., 2015a,b) were of high quality [Refer to tables, studies are indicated with an (‡)]. Fifteen of the studies referenced loaded stretch positions with eight having the participants stretch to discomfort (McNair and Stanley, 1996; Hayes and Walker, 2007; Curry et al., 2009; Rancour et al., 2009; Maluf et al., 2010; Silveira et al., 2011; Cipriani et al., 2012; Wicke et al., 2014), one to pain (Smith et al., 1993), five to MSNP (Muir et al., 1999; de Weijer et al., 2003; Youdas et al., 2003; Horsley et al., 2007; Borman et al., 2011), with the last study having participants stretch to a gentle stretch intensity (O'sullivan et al., 2009). One study had participants stretched to pain with use of a therapist (Apostolopoulos et al., 2015a). The majority of the studies referencing discomfort, pain and MSNP presented conflicting results as to whether stretching was beneficial. However, it is interesting to note that the gentle loaded stretch (O'sullivan et al., 2009) compared to the remaining six high quality studies, which referenced supported gentle stretches (Zakas et al., 2006; Wyon et al., 2013), discomfort with use of a therapist (Cristopoliski et al., 2009) or machine (Medeiros et al., 1977; Apostolopoulos et al., 2015b), and a MSNP with a therapist (Clark et al., 1999), all indicated that stretching under these conditions was beneficial. The study by Apostolopoulos et al. (2015b) comparing various stretch intensities based on an individual's maximum ROM (mROM) concluded that stretches between 30 and 60% mROM (gentle stretch intensity) did not cause inflammation, whereas a stretch of 90% mROM (pain) caused inflammation.

A study which had dancers perform either a gentle stretch or a discomfort intense stretch in a supported position; observed for the gentle intense supported stretch produced the greatest gains in terms of increasing active and passive ROM (Wyon et al., 2013). It is likely that the gentle supported stretch position influenced the series and parallel elastic components, and thereby prevented the activation of a stretch reflex. When a relaxed muscle is stretched, the change in length is shared between the series and parallel elastic components (Buller, 1975). In contrast, when a muscle contracts isometrically, the parallel elastic element is unloaded, while the series elastic element is stretched by an amount dependent on the force developed to the muscle. The activation of the series elastic element stimulates the Golgi Tendon Organ, since this is in series with this component (Kandel et al., 2000). The increase in muscle tension during activation may be a by-product of a loaded stretch in conjunction with an intensity level perceived as a discomfort or pain. This indicates the potential importance of the effect of the force generated during stretching exercises, as well as the position assumed by the participant during the actual stretching exercise.

The elongation of passive muscle from short to medium lengths requires little application of force. However, stretching muscle to greater lengths requires a large force (Jespersen, 1950). The force generated during a single intense stretch has been associated with several outcomes: overt skeletal muscle injury (inflammation, myofiber degeneration, and dysfunction), skeletal muscle adaptation (regeneration and growth with functional gains), and/or mal-adaptation (a sub-degenerative or sub-necrotic state that is usually associated with low levels of persistent inflammation and loss of function) (Cutlip et al., 2009). The intensity of the stretch during a stretching exercise has been described as the magnitude of the force, and it has been suggested that if the force applied is too much this may injure the tissue resulting in an inflammatory response (Brand, 1984; McClure et al., 1994). In the study conducted by Light et al. it was observed that use of a low-load prolonged stretch was found to be superior to a high-load brief stretch in treating knee contractures in 11 elderly patients. Therefore, this study may suggest that a low intense passive stretch held at a constant length may lead to a stress relaxation. According to Kubo et al. (2001), a stretch being held at a constant length influences the MTU resulting in a reduction in the stiffness which may be responsible in the increase in the joint ROM. Therefore, the magnitude of the force applied during the stretch may influence acute and chronic ROM.

As previously highlighted, the body's response to this force is to activate components of the immune system which, depending on the severity of the response, may result in significant impairment (Cuthbertson, 1942). Chronic exposure to high force development has been shown to create inflammatory manifestations (Archambault et al., 2001; Stauber and Willems, 2002; Barbe and Barr, 2006). Such mechanical loading, associated with the overstretching of sarcomeres beyond the myofilament overlap, creates a physical disruption of the musculoskeletal fibers, resulting in pain and inflammation (Gregory et al., 2002). In turn, the activated local pathways of the damaged tissue further mediates inflammation and tissue damage (Armstrong et al., 1983; Fridén et al., 1986; Geronilla et al., 2003).

## Conclusion

This systematic review reveals that only a few of the published papers in this area of research examined articles addressing the intensity of stretching, even across a variety of population groups. This component of stretching may be linked to increased inflammation in chronic conditions when stretching is used to deal with clinical conditions or improving the ROM of soft and connective tissue in both therapeutic and athletic environments. In addition, due to the lack of good quality studies, it is difficult to draw conclusions about the effects of stretching intensity and/or position on the observed effects of stretching. More research is required concerning the appropriate application of stretching intensity, and the critical role it might play in optimizing musculoskeletal health. What is needed is a high quality study perhaps using a new research paradigm. This study would examine the efficiency of a low intense supported stretch as a means to increasing ROM, without setting off the stretch reflex, while minimizing inflammation.

### Conflict of interest statement

The authors declare that the research was conducted in the absence of any commercial or financial relationships that could be construed as a potential conflict of interest.
